# Draft genome sequences of five *Mycobacterium* strains, isolated from *Alnus glutinosa* root nodules

**DOI:** 10.1128/mra.01132-23

**Published:** 2024-01-08

**Authors:** Ryan Michael Thompson, Edward M. Fox, Maria del Carmen Montero-Calasanz

**Affiliations:** 1 School of Natural and Environmental Sciences, Newcastle University, Newcastle upon Tyne, United Kingdom; 2 Department of Applied Sciences, Northumbria University, Newcastle upon Tyne, United Kingdom; 3 IFAPA Las Torres-Andalusian Institute of Agricultural and Fisheries Research and Training, Junta de Andalucía, Seville, Spain; Rochester Institute of Technology, Rochester, United States

**Keywords:** *Mycobacterium*, endophytes, *Alnus glutinosa*, *Alnus*, genomes

## Abstract

*Mycobacterium* is a clinically relevant genus of bacteria, with this paper reporting draft genomes of five *Mycobacterium* strains derived from *Alnus glutinosa* root nodules. The genome sizes of the isolates ranged from 6.1 to 6.9 Mbp, composed of 22–59 contigs. The *N*_50_ values ranged from 303,875 to 865,751 bp, presenting a GC% of 66.07%–66.96%.

## ANNOUNCEMENT

*Mycobacterium* is a clinically important genus, including notable pathogens such as *Mycobacterium tuberculosis*. Aside from these, mycobacteria are well-documented plant endophytes, with five *Mycobacterium* isolated from the root nodules of *Alnus glutinosa* during this investigation (1, 2). *A. glutinosa* is of interest in the field of bioremediation; however, its root microbiome remains understudied with the roles of many bacteria unknown, so this niche was selected for further investigation (3–5).

*A. glutinosa* nodules were collected from a single tree within Saltwell Park, Gateshead, UK (54.944723, –1.605852). The nodules were washed with tap water, with 4–15 nodule lobes removed and surface sterilized for 5 minutes in 1 mL of 25% strength household bleach (~1.125% sodium hypochlorite). The bleach was removed, and the lobes were washed five times for 5 minutes in 1 mL of sterile distilled water, and then homogenized in 0.5 mL of 1/4 strength Ringers solution. To this homogenate, 14.5 mL of 1/4 strength Ringers solution was added, with this plated upon basic propionate (BAP) agar (https://www.dsmz.de/microorganisms/medium/pdf/DSMZ_Medium1536.pdf), alkaline BAP agar (pH 9), and Zhang starch soil extract agar (6) (all media containing 50 mg/L nystatin), with all plates incubated at 28°C. All isolates were recovered after 30 days of incubation, with RTGN3 recovered from Zhang starch soil extract agar, RTGN4, RTGN6, and RTGN8 recovered from alkaline BAP agar, and RTGN5 recovered from BAP agar. Axenic cultures were developed by streaking a single colony upon each respective medium and incubating the plates for 24 days at 28°C, except in the case of RTGN4, which was incubated for only 10 days. Three iterations of subculturing were conducted from a single colony to achieve an axenic culture.

DNA was extracted from 4-day-old axenic material of RTGN3 and RTGN4, 10 days old in the case of RTGN5 and RTGN8, and 2 days old in the case of RTGN6. Extractions were performed using a GenElute Bacterial Genomic DNA Kit (Sigma-Aldrich, USA) with an additional ethanol precipitation. Sequencing was conducted by Novogene Co. Ltd. with a DNA library prepared using a Novogene NGS DNA Library Prep Set (Cat No. Pt004), in which the DNA was randomly sheared into short fragments, end repaired, A-tailed, and then ligated with the Illumina adaptor. These sequences were amplified using PCR, size selected for 350 bp, purified, and then sequenced using 150 bp Illumina paired-end sequencing upon an Illumina NovaSeq. The reads were filtered using FastP (version 0.23.1) to remove adapter sequences, followed by reads that contained more than 10% uncertain nucleotides or more than 50% low-quality reads (base quality < 5).

All subsequent steps were conducted using the default settings unless otherwise noted. All filtered reads were uploaded to Galaxy Europe (https://usegalaxy.eu/) (7) (Table 1), assessed using FASTQC (Galaxy Version 0.73 + galaxy0) (http://www.bioinformatics.babraham.ac.uk/projects/fastqc/), assembled using Shovill (Galaxy Version 1.1.0 + galaxy1) (https://github.com/tseemann/shovill), and the assemblies were assessed using Quast (Galaxy Version 5.0.2 + galaxy5), with contigs < 200 bp removed (8–10) (Table 1). The relationship of the isolates to their nearest neighbors was determined using the TYGS webserver (v389) (11, 12) (Table 1).

**TABLE 1:** Read, assembly statistics, and nearest neighbors for all isolates

Isolate	Raw read number	Filtered read number	Assembly length (bp)	Contig number	*N*_50_ value	GC%	Estimated sequencing depth	Nearest neighbor (percentage identity)
RTGN3	10,889,178	10,872,052	6,914,315	35	399,931	66.96	236	*Mycobacterium sediminis* JCM 17899 (21.4)
RTGN4	11,909,150	11,895,184	6,859,201	58	409,151	66.06	260	*Mycobacterium madagascariense* JCM 13574 (21.4)
RTGN5	11,395,678	11,380,462	6,125,641	22	865,751	66.42	279	*Mycobacterium helvum* JCM 30396 (35.3)
RTGN6	12,087,980	12,072,066	6,913,698	31	425,402	66.96	262	*Mycobacterium sediminis* JCM 17899 (21.4)
RTGN8	11,634,726	11,620,034	6,860,099	59	303,875	66.07	254	*Mycobacterium madagascariense* JCM 13574 (21.5)

## Data Availability

The draft genomes of RTGN3, RTGN4, RTGN5, RTGN6, and RTGN8 are available within INSDC under the accession numbers JAPZLC000000000, JAPZLB000000000, JAPZLA000000000, JAPZKZ000000000, and JAPZKY000000000, respectively. The SRA data of RTGN3, RTGN4, RTGN5, RTGN6, and RTGN8 are available within INSDC under the accession numbers SRR22064634, SRR22064993, SRR22065103, SRR22065910, and SRR22066022, respectively.
